# Acetazolamide: Treatment of Psychogenic Polydipsia

**DOI:** 10.7759/cureus.1553

**Published:** 2017-08-09

**Authors:** Syed E Ahmed, Afaque H Khan

**Affiliations:** 1 Heartland Behavioral Healthcare, Department of Mental Health and Addiction, State of Ohio, Northeast Ohio Medical University; 2 Psychiatry, Heartland Behavioral Healthcare, Department of Mental Health and Addiction, State of Ohio, Northeast Ohio Medical University

**Keywords:** psychogenic polydipsia, water intoxication, schizoaffective disorder bipolar type

## Abstract

We are reporting a case of psychogenic polydipsia from a State of Ohio psychiatric hospital. The patient has a known five-year history of psychogenic polydipsia with recurrent hyponatremia and has been diagnosed with schizoaffective disorder bipolar type 1, according to the Diagnostic and Statistical Manual of Mental Disorders, Fifth Edition (DSM-5) criteria, for the past two decades. There was a marked improvement with the use of acetazolamide, resulting in a decreased compulsion to drink fluid and improvement of his hyponatremia. The patient was observed for six months. We evaluated the water balance of the patient with diurnal weight measurements (DWG) and a weekly comprehensive metabolic panel (CMP) to monitor Na⁺ levels. His symptoms and hyponatremia were improved with acetazolamide. The treatment was well tolerated without any adverse effects and improved his quality of life.

## Introduction

Acetazolamide, a carbonic anhydrase inhibitor, is mostly used as a diuretic; however, it is also used for various conditions, including glaucoma, epilepsy, intracranial hypertension, and acclimatization. This agent inhibits bicarbonate (HCO₃) reabsorption in the proximal tubules of the kidneys and leads to HCO₃ wasting by inhibiting the carbonic anhydrase, consequently causing metabolic acidosis. It decreases intracellular Na⁺ concentration and the rate of Na⁺ turnover. There are various hypotheses about the mechanism of polydipsia; once such mechanism describes disturbances in ATII (angiotensin II) secondary to hypersensitivity to the D2 receptor or dysfunctions of the D4 receptor. However, the mechanism of polydipsia in patients with chronic psychiatric illness is poorly understood and, therefore, difficult to manage. The mechanism of acetazolamide on D2 and D4 receptors, as well as ATII, is poorly understood [1].

## Case presentation

The patient is a 56-year-old Caucasian male who has been an inpatient at a state psychiatric hospital for the past 18 years. The patient has been residing at various state-run psychiatric hospitals for past 33 years. He was diagnosed with a schizoaffective disorder in 1976 at the age of 15. His symptoms include auditory hallucinations, loose association, disorganized thought process, rapid speech, and self-injurious behavior. He has had a history of psychogenic polydipsia for the past five years. He demonstrated symptoms of repeated compulsive fluid intake, resisted restriction of fluid intake, and required restriction of movement outside the unit depending upon the severity of polydipsia or water intoxication. Also, weight gain was evaluated in terms of normalized diurnal weight gain (NDWG), which is the percentage increase in weight from morning to evening. During episodes of polydipsia, no sign of severe water intoxication, such as confusion, delirium, seizures, or coma, were observed. His vital signs were stable with each episode. The patient was given trials of ACE inhibitors and second generation antipsychotics like clozapine, but these were discontinued due to ineffectiveness and undesirable side effects. These included significant incidence of agranulocytosis and orthostatic hypotension with clozapine [1] and electrolyte disturbances with ACE inhibitors. Therefore, another pharmacological strategy was warranted. The decision was made to initiate a therapy of acetazolamide. Informed consent was obtained from both the patient and his guardian before starting medication. Fluid restriction had been the key treatment of PPD; however, this did not resolve the polydipsia, and often the treatment was time-consuming and difficult. There is evidence that both the conditions, psychogenic polydipsia and water intoxication, are resistant to several pharmacological treatments. If this condition is not resolved completely, it can lead to deterioration both mentally and functionally and a prolonged hospital stay (Table 1).

**Table 1:** Management with Water Restriction Only Prior to Acetazolamide Treatment Normal Na level: 136-145 mEq/L; Patient target weight gain < 5 lbs. DWG: diurnal weight measurements; WI: water intoxication

Month	Polydipsia	Weight gain lbs. (DWG)	Na, mEq/L	WI	Movement on hold
1	severe	+4.5 - 6.5	130 - 135	sometimes	yes
2	severe	+4.5 - 6.5	128 - 135	sometimes	yes
3	severe	+4.5 - 6.5	130 - 134	sometimes	yes

The patient showed a significant response to the acetazolamide. He was started on a daily dose of 250 mg. There was an improvement in hyponatremia and polydipsia, as well as water intoxication. His weight was stable and no diurnal weight gain was noted or observed. The patient was able to utilize all his privileges without restriction or limitation. A strong negative correlation between hyponatremia and water intoxication was observed. The patient has tolerated the medication well without side effects on at his three-month follow-up (Table 2).

**Table 2:** After Starting Acetazolamide, 250 mg qd DWG: diurnal weight measurements; WI: water intoxication

Month	Polydipsia	Weight gain lbs. (DWG)	Na, mEq/L	WI	Movement on hold
1	mild	+3.5 - 4	131 - 135	none	no
2	mild	+3 - 3.5	131 - 137	none	no
3	none	+2 - 2.5	136 - 138	none	no

## Discussion

Psychogenic polydipsia (PPD) is a well-recognized condition that frequently occurs secondary to chronic mental illness, particularly schizoaffective disorders and schizophrenia. This condition manifests as an excessive compulsive fluid intake without any underlying medical cause. Occasionally, PPD presents concurrently with hyponatremia, which may cause neurological symptoms. Symptoms range from headache, vomiting, and lethargy to psychosis and seizures and may even be fatal [2]. These manifestations are secondary to acute cerebral edema caused by sudden or severe hyponatremia and decreased free water clearance.

Fluid restriction has been the key treatment of PPD; however, this decreases only the risk of water intoxication but does not resolve the polydipsia. Often, the treatment is also time-consuming and difficult. If this condition is not resolved completely, it can lead to deterioration both mentally and functionally and can lengthen hospital stays. Fluid restriction has no influence on psychogenic polydipsia; therefore, pharmacological intervention is preferred. The decision was made to initiate treatment with acetazolamide. There was a significant improvement in hyponatremia and polydipsia. Acetazolamide, initially used as a diuretic, inhibits bicarbonate reabsorption in proximal tubules by inhibiting carbonic anhydrase and leads to metabolic acidosis from bicarbonate wasting in the kidney tubules. It reduces intracellular sodium concentration. Also, acetazolamide acts in the proximal tubules to decrease the reabsorption of sodium, although most of the sodium reabsorbs in the distal tubules. It has a mild natriuretic effect and the adverse effect of hyponatremia [1]. In our case report, the improvement in excessive fluid intake behavior and hyponatremia indicates that acetazolamide works on the central nervous system and kidneys.

## Conclusions

Psychogenic polydipsia in patients with chronic psychiatric illness is often difficult to manage and can be fatal if left untreated. It is characterized by compulsive consumption of large quantities of fluid, which results in “dilutional hyponatremia” or low plasma sodium. Recently, we had success in treating a patient with psychogenic polydipsia with acetazolamide (carbonic anhydrase inhibitor). We found improvement in his compulsive fluid intake behavior and hyponatremia. In addition to medication, water restriction and daily weight monitoring were key tools in managing the PPD. This case report demonstrates the beneficial effects of acetazolamide in managing psychogenic polydipsia and hyponatremia.
